# Editorial: Interfacial Water: A Physical Chemistry Perspective, Volume II

**DOI:** 10.3389/fchem.2022.896586

**Published:** 2022-05-23

**Authors:** Yoshihisa Harada

**Affiliations:** Institute for Solid State Physics, The University of Tokyo, Kashiwa, Japan

**Keywords:** interfacial water, hydration, aquatic functional materials, surface and interface, hydrogen bonding

Water’s properties have always been studied using cutting-edge analytical methods, which is critical because water comprises the earth’s surface and our daily lives. However, even the structure of bulk water is being debated, so when it comes to interfacial water, things become much more complicated due to the variety of water networks and interacting materials (Brini et al., 2017). While interfacial water contributes to the diversity of material functions, each phenomenon is so distinct that developing a general concept of interfacial water appears challenging. In the Research Topic on *Interfacial Water: A Physical Chemistry Perspective* , a wide range of viewpoints are used to examine the properties of interfacial water, both from the perspective of water and materials. Because of the success of this Research Topic, Volume II was published, which advances the study of interfacial water by including a broader range of analytical approaches capable of capturing water structure and dynamics. Furthermore, the materials covered are diverse, ranging from solid surfaces to proteins.

In terms of the nature of interfacial water, phospholipid bilayer is a well-studied material. Scollo et al. demonstrated that the degree of hydration and mobility of the interfacial region formed by hydrated lipid headgroups, as well as the composition of the bilayer and headgroups, can be visualized in images using Time-Dependent Fluorescence Shift. They discuss how ions and oxidized phospholipids affect bilayer organization and headgroup packing. Bonini et al. used Small Angle Neutron Scattering and Elastic Neutron Scattering to study the aging of Bio Crude Oils, potential renewable energy sources, and their lignin fractions to better understand aggregate growth and fractal hierarchy. The quantitative discussion of the temporal and spatial changes in molecular activity employs Quasi-Elastic Neutron Scattering to capture the formation and then the dynamics changes.

Lautenbach et al. used sum frequency generation spectroscopy to quantify the polarity and magnitude of the electric field in the hydration shell of a specific model protein adsorbed on a hydrophobic surface, polystyrene (PS). It was discovered that the protein’s pH-dependent adsorption behavior on PS correlates with its amino acid composition and degree of hydrophobicity. Meanwhile, Nakagawa et al. investigated the hydration structure and hydrogen bonding states of the protein staphylococcal nuclease at various hydration levels in the crystalline state using all-atom molecular dynamics (MD) simulations. They discovered that the distance and angle of hydrogen bonds in the crystal’s hydration water were comparable to those of tetrahedrally-coordinated water molecules in the bulk.

To precisely characterize the character and functional role of water at well-defined surfaces, various interface-sensitive techniques have been used; Li et al. used an external gate potential in conjunction with an electrolyte-insulator-semiconductor junction to control the charge density and charge strength at the silica/water interface. Sum-frequency vibrational spectroscopy was used to successfully observe the alignment of liquid water molecules. Cao et al. reviewed the use of atomic force microscopy (AFM) to observe two-dimensional (2D) or three-dimensional (3D) interfacial water at a variety of solid surfaces in order to gain a better understanding of its physicochemical properties. Chang et al. used the AFM to measure surface forces precisely. Antifouling properties of specific zwitterionic amino acid sequences is found to differ significantly, and interfacial water in the vicinity of the specific amino acid sequences may act as a physical barrier to prevent protein and cell adsorption or adhesion. Fujii et al. used Quasielastic Neutron Scattering to observe hydrated poly (methyl methacrylate) (PMMA) and discovered that hydrated water affects the local motion of PMMA and activates local relaxation processes. Akada et al. used soft X-ray emission spectroscopy to observe hydrogen-bonded structure of water in a loop-shaped poly (ethylene glycol) chain in a polyrotaxane and discovered the lack of tetrahedrally-coordinated water in the loop possibly due to entropy loss, suggesting the ability to control the biocompatibility.

It is clear from the preceding investigations that interactions between water and materials alter their respective structures and dynamics, as well as intricately control the function at the interface. Modern analytical methods are being developed on a more microscopic scale, with higher time resolution, or with increased chemical state or interface selectivity, and are expected to shift away from typical interface analysis and toward practical materials analysis in the near future.
